# Supplementary material for: Identification of global inhibitors of cellular glycosylation
Source: Nat Commun. 2023 Feb 20;14:948. doi: 10.1038/s41467-023-36598-7 (PMC9941569; doi:10.1038/s41467-023-36598-7)
Supplement: Supplementary file 1 — Supplementary Information [file 41467_2023_36598_MOESM1_ESM.pdf]

Supplementary Information

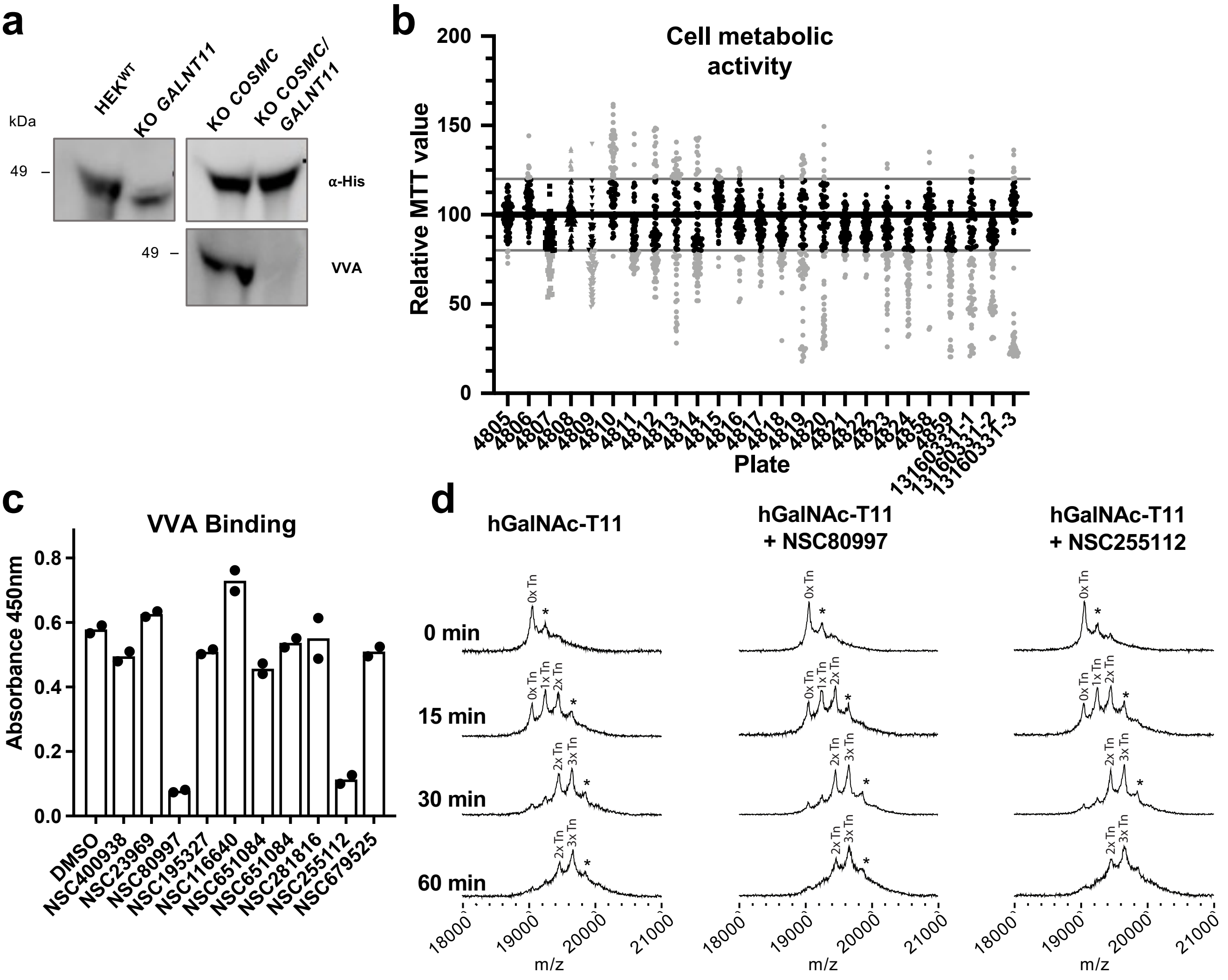

Supplementary Figure 1 A) Representative western blot shows change in molecular weight of the LDLR reporter secreted from HEK293^{WT}, HEK293$^{KO\ \mathit{GALNT11}}$, HEK293$^{KO\ \mathit{COSMC}}$ and HEK293$^{KO\ \mathit{COSMC/GALNT11}}$ cells. Reporters were detected with anti-6xHis-tag antibody and Tn epitope was detected by VVA lectin. **B)** Dot plot shows metabolic activity of HEK293$^{KO\ \mathit{COSMC}}$ $^{+LDLR}$ reporter cells treated 24 h with 1952 compounds. Data points are organized per 96-well plate, each containing 80 compounds and 3 DMSO controls. MTT absorbance is presented as fold change normalized to DMSO control (black line). Data points were excluded from further analysis if cell metabolic activity deviated by more than 20% from DMSO control (grey lines). **C**) Bar diagram shows binding of VVA to captured LDLR reporter protein secreted by HEK293$^{KO\ \mathit{COSMC}}$ cells treated with select compounds for 24 h. Average absorbance values of two independent ELLA experiments are shown. **D)** Time course *in-vitro* glycosylation of LDLR LA1-4 linkers by recombinant GalNAc-T11 in the absence or presence of 50 µM NSC80997 or NSC255112, evaluated by MALDI-TOF-MS. Mass peak corresponding to non-glycosylated LDLR LA1-4 liker is presented as 0xTn, and peaks corresponding to addition of N-aceylgalactosamine being labelled n xTn. *Contaminant

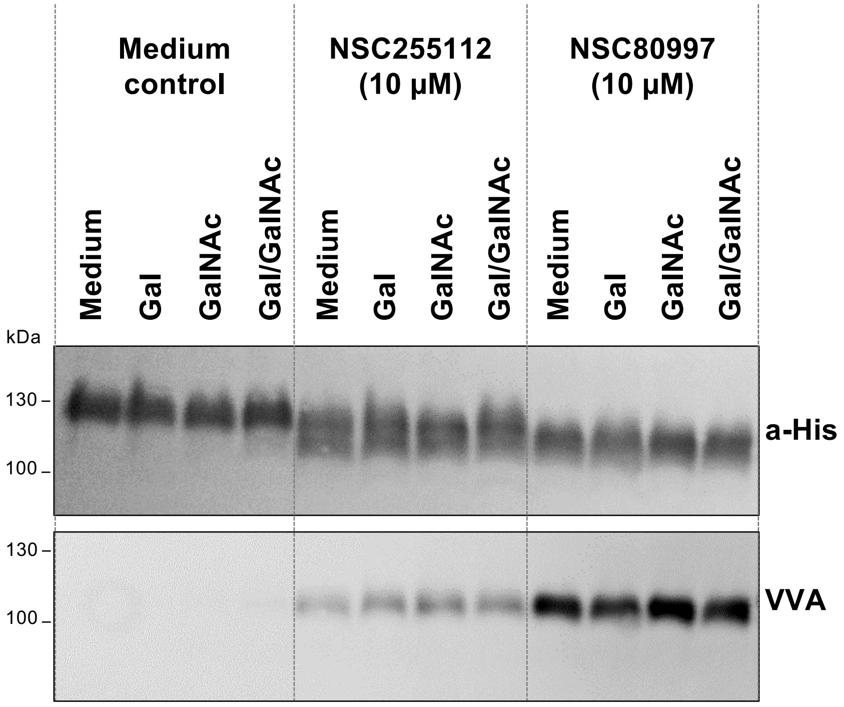

Supplementary Figure 2 HEK293^{WT} cells stably secreting the MUC1 O-glycosylation reporter were incubated 24 h with 10 µM NSC255112 or NSC80997 in culture media supplemented with either 1 mM galactose (Gal), 10 mM GalNAc, or the combination of both. Representative western blot shows MUC1 reporter protein from culture supernatants detected by anti-6xHis-tag antibody (upper panel) and VVA (lower panel).

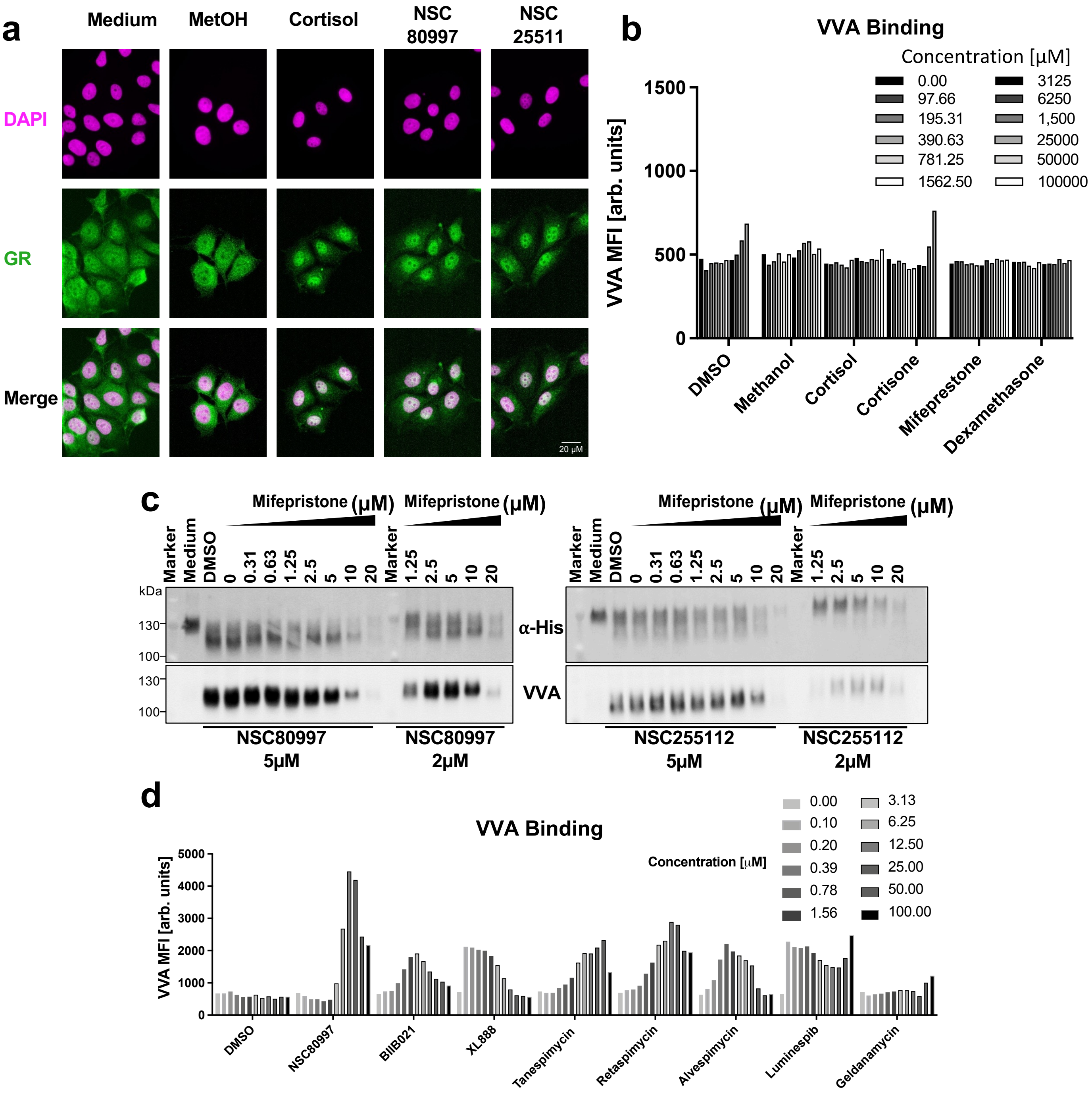

Supplementary Figure 3 A) Representative images of MCF7^{WT} cells treated with 10 µM cortisol, NSC80997 or NSC255112 for 90 min and intracellularly stained with anti-glucocorticoid receptor (GR) antibody (green) and DAPI (magenta). Cells treated with equal volumes of medium and methanol (MetOH) are shown as control. Scale bars represent 20 µm **B)** Bar diagram shows cell surface VVA binding to HEK293^{WT} cells treated 24 h with increasing concentrations of mifepristone, cortisone, cortisol, and dexamethasone. DMSO and methanol served as controls. VVA binding was assessed by flow cytometry, is presented as mean fluorescence intensity (MFI) (arbitrary units) and is representative of two independent experiments. **C)** HEK293^{WT} cells stably secreting MUC1 O-glycosylation reporter were incubated 24 h with 5 µM or 2 µM NSC80997 or NSC255112 together with 0-20 µM Mifepristone. Representative western blot shows MUC1 reporter protein detected by anti-6xHis-tag antibody (upper panel) and VVA (lower panel). **D)** Bar diagram shows cell surface VVA binding to HEK293^{WT} cells treated 24 h with increasing concentrations of DMSO control, NSC80997, BIIB021, XL888, tanespimycin, retaspimycin, alvespimycin, or luminespib. VVA binding was assessed by flow cytometry, is presented as mean fluorescence intensity (MFI) (arbitrary units) and is representative of two independent experiments.

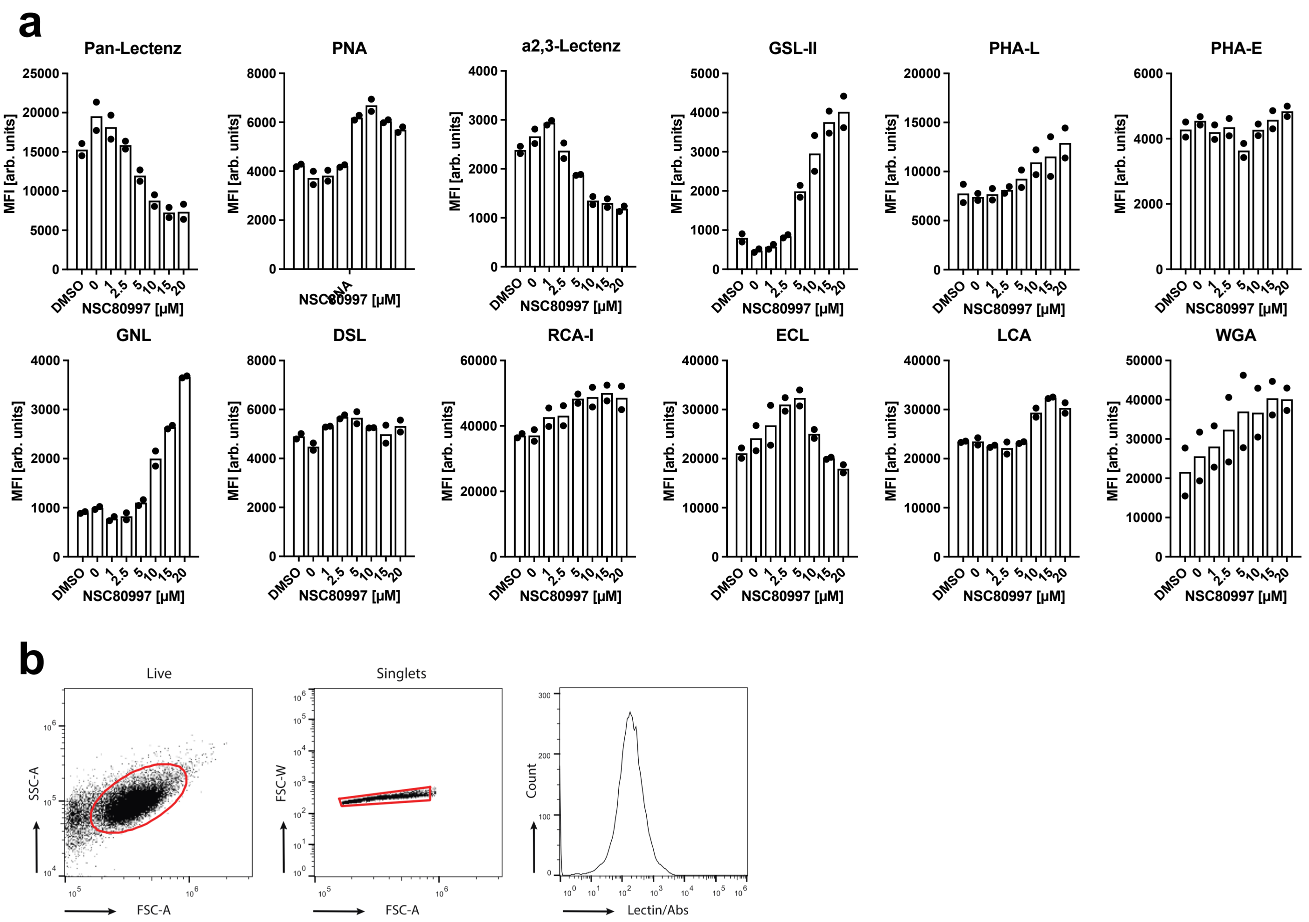

Supplementary Figure 4 A) Bar diagrams show cell surface glycosylation profiling of HEK293^{WT} cells with lectins. Cells were treated with increasing concentrations of NSC80997, or DMSO control for 24 h and lectin binding was assessed by flow cytometry. Data from two independent experiments are presented as average mean fluorescence intensity (MFI) (arbitrary units). **B**) Gating strategy to measure lectin or monoclonal antibody binding to cells. Dead cells were excluded based on forward and side scatter area (FSC-A and SSC-A) parameters. Doublets were excluded based on FSC-A and FSC-W (width) parameters

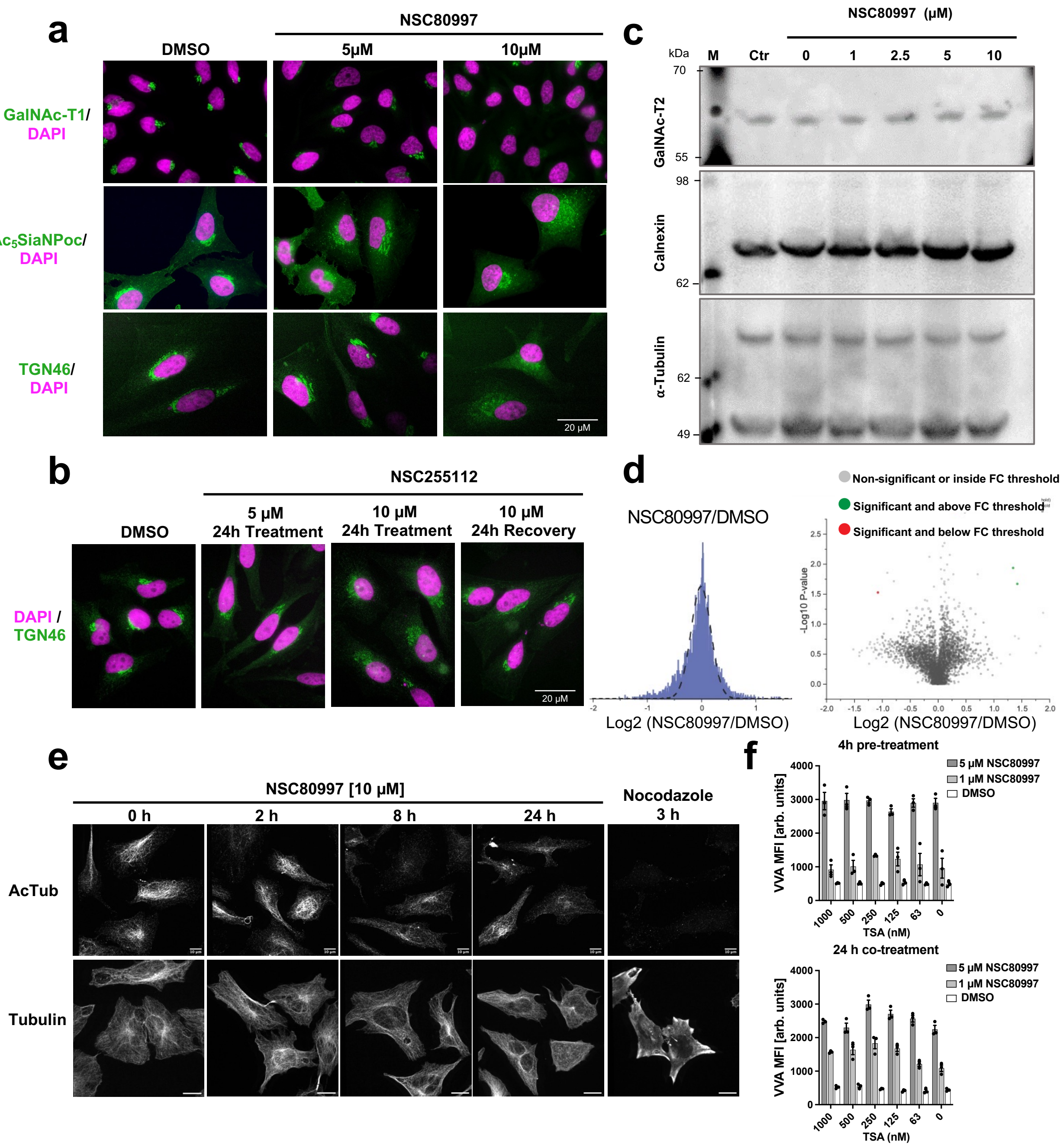

Supplementary Figure 5 A) Representative images of HeLa^{WT} cells treated with DMSO or 5 µM and 10 µM of NSC80997 for 24 h stained for Golgi markers with anti-GALNT1 (mAb 4D8) (*cis*-Golgi), anti-TGN46 (*trans*-Golgi), or sialoglycans metabolically labelled by Ac$_5$SiaNPoc (medial/*trans*-Golgi). Nuclei staining by DAPI is shown (magenta). Scale bar represents 20 µm **B)** Representative images of Golgi fragmentation and re-organization in HeLa^{WT} cells treated with 5 µM and 10 µM NSC255112 for 24 h, or cells that were washed and re-cultured for 24 h without treatment. Staining for TGN46 (green), and DAPI (magenta) is shown. Scale bar represents 20 µm **C)** Representative western blots detecting GALNT2 (top), calnexin (middle), and α-tubulin (bottom, lower specific band) in total cell lysates of HEK293^{WT} cells treated with increasing concentrations (0-10µM) of NSC80997 for 24 h are shown. Molecular weight marker (M) is indicated **D)** Differential proteome analysis comparing HEK293WT cells treated with 10 µM NSC80997 or DMSO control for 24 h. Histogram shows the distribution of NSC80997/DMSO protein ratios for TMT-labelled peptides from total cell lysate (left). Volcano plot depicts fold changes of identified proteins. Fold change threshold of 2 and adjusted p-value (t-test) of 0.05 were used to identify differentially expressed proteins in NSC80997 cells against DMSO control. P-values were adjusted using the Benjamini-Hochberg method (right). **E)** Representative images of HeLa^{WT} cells treated with 10 µM NSC80997 or 33 µM Nocodazole stained for acetylated tubulin (top) or tubulin (bottom) at different time points. Scale bars represent 10 µm. **F)** Bar diagrams shows cell surface VVA binding to HEK293^{WT} cells pre-treated 4 h with increasing concentrations of trichostatinA (TSA) (0-1000 nM) before 24 h treatment with 5 µM or 1 µM NSC80997 or DMSO (top) or co-treated with corresponding concentrations of TSA and NSC80997 for 24 h. Binding was measured by flow cytometry and data is presented as mean MFI ± SEM (arbitrary units) from three independent experiments.

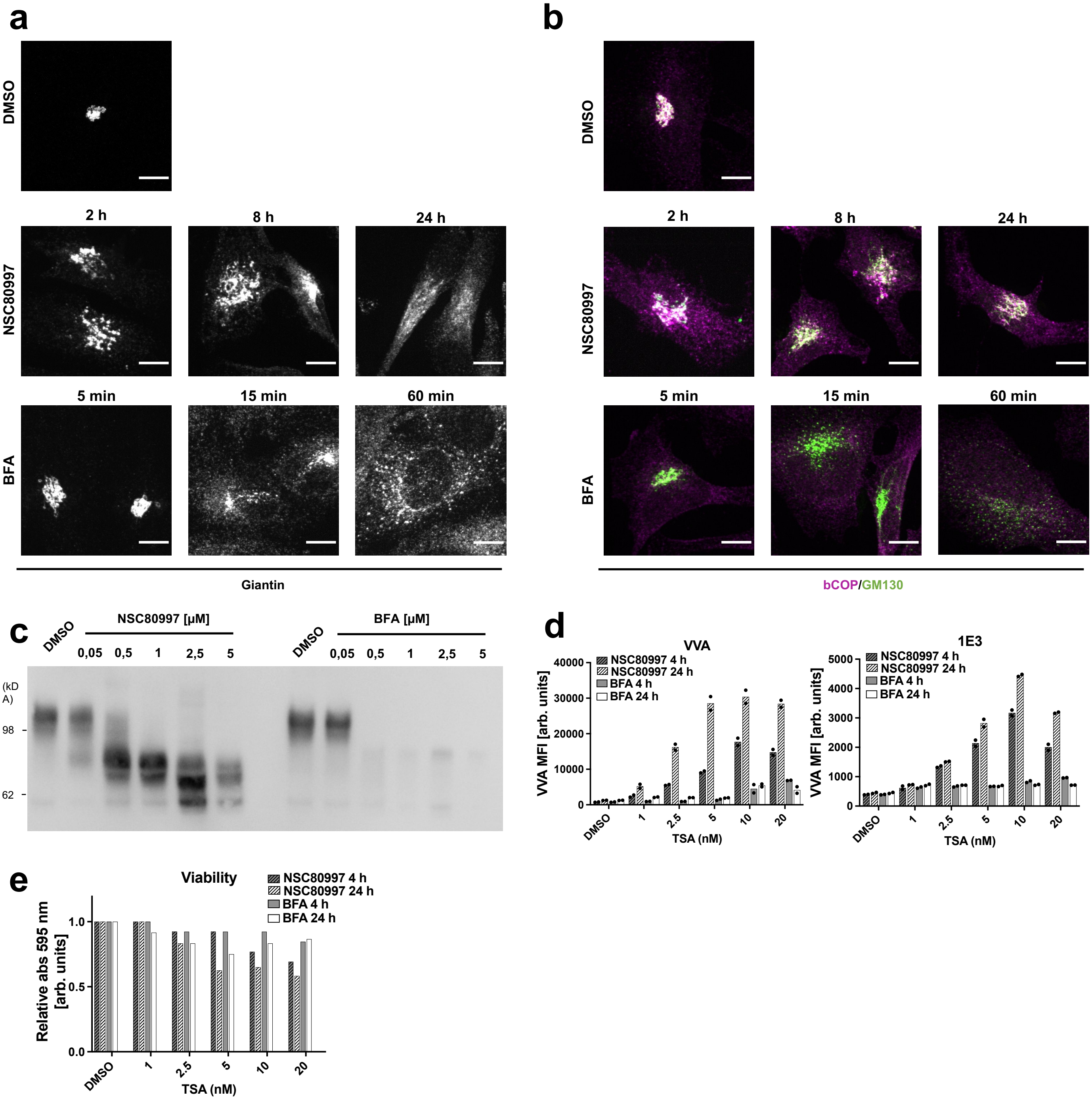

Supplementary Figure 6 A-B) Representative images of HeLa^{WT} cells treated with DMSO, 10 µM NSC80997 or BFA and stained for Giantin **(A)** or bCOP and GM130 **(B)** at different time points. Scale bars represent 10 µm. **C)** Representative western blot shows the MUC1 reporter produced in HEK^{WT} cells incubated with increasing concentrations of NSC80997 or Brefeldin-A (BFA) and detected by anti-6xHis-tag antibody. Cells were pretreated for 4 h with corresponding compound concentration, washed to remove any reporter produced prior to treatment and incubated for 24 h in the presence of compound. **D)** Bar diagrams shows cell surface VVA and 1E3 binding to HEK293^{WT} cells treated 4 h or 24 h with increasing concentrations of NSC80997 or BFA or DMSO control. Binding was measured by flow cytometry 24 h after treatment initiation and data is presented as average MFI (arbitrary units) from two replicates. **E)** Representative bar diagram shows metabolic activity of HEK^{WT} cells treated with 1-20 µM NSC80997 or DMSO treatment for 4 h or 24 h measured by MTT assay. Data is presented as relative 595 nm absorbance values (arbitrary units) compared to DMSO and is representative of two independent experiments.

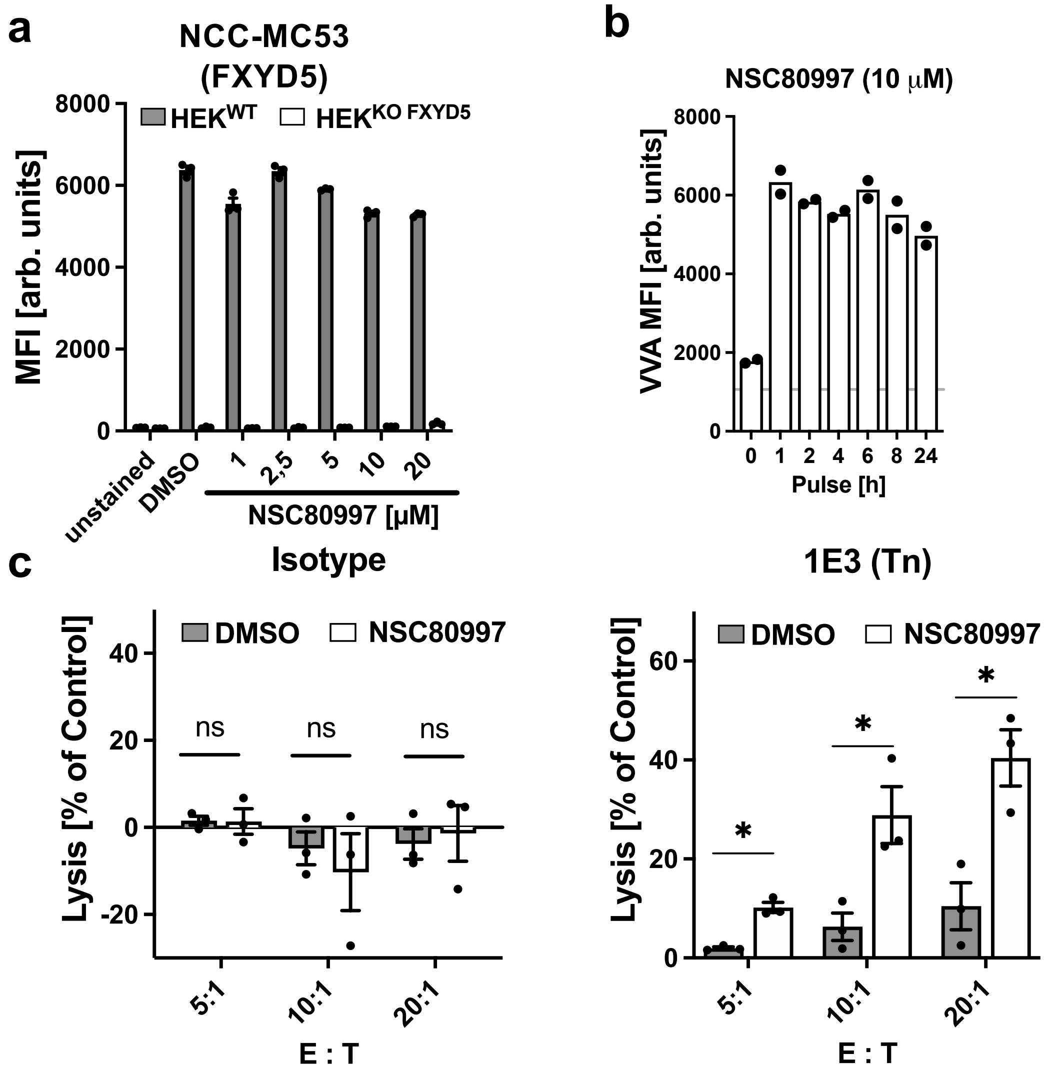

Supplementary Figure 7 A) Cell surface binding of NCC-MC53 (FXYD5) to HEK^{WT} and $HEK^{KO\ FXYD5}$ cells treated with increasing concentrations of NSC80997 for 24 h. Antibody binding was measured by flow cytometry and data is shown as mean MFI ± SEM (arbitrary units) of three independent experiments. **B)** $HEK293^{WT}$ cells were pulsed with 10 µM NSC80997 for 0-24 h, washed, and subsequently incubated in fresh culture media for 24 h. VVA binding was assessed by flow cytometry and is presented as average MFI (arbitrary units) from two independent experiments. **C)** ADCC assay with $HEK293^{MUC1}$ cells (targets) pulsed for 4 h with DMSO or 10 µM NSC80997 and co-cultured overnight with different ratios of human PBMCs (effectors) in the presence of isotype or 1E3 monoclonal antibody. Data is presented as mean percentage lysis compared to control ± SEM from 3 independent experiments. *$P < 0.05$, ^{ns} not significant (t-test).

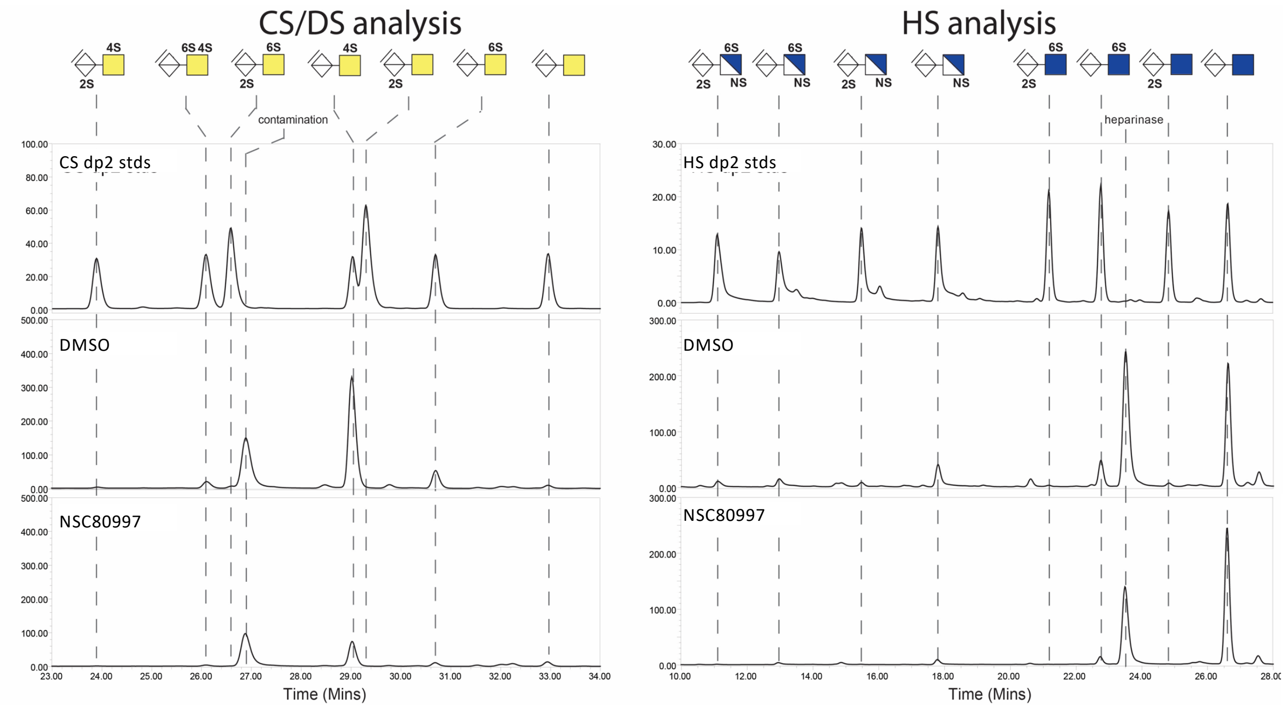

Supplementary Figure 8) HPLC disaccharide analysis of CS/DS and HS from total cell lysates of HEK293 cells treated with NSC80997 or DMSO as control for 24 h. The top chromatograms show disaccharide standards (20 picomoles of each standard). Equal amounts of cells were used for extraction of GAGs for all samples, and chromatograms are representative of 3 independent experiments.

HEK293 Engineered cells	Target exon	gRNA sequence	Forward primer (5’-3’)	Reverse primer (5’-3’)	In-del (5’-3’)
ΔGR (NR3C1)	2	AGTTGTCATCTCCAGATCCT	GAGTCCCCAGAGAAGTCAAG	AGCAAGAGAAACTGGGCAC	+1: AGATCCTTGGCACCATATTC
ΔAR (NR3C4)	1	CCGCCGTCCAAGACCTACCG	ATGGAAGTGCAGTTAGGGCTG	CTTGGGGAGAACCATCCTCA	+1: GACCTACCGAGGAGCGTTTCC

Supplementary Table 1) List of CRISPR gRNA designs and PCR primers introduced in this study, and the resulting In-del sequences in the studied cell clones. Nucleic acids in Red are the insertion or deletion. Nucleic acids in Blue are the PAM sequence

Chemical supplementary information

General procedures: ^{1}H and ^{13}C NMR spectra were recorded on a Bruker Avance III 500 MHz spectrometer. Chemical shifts are reported in parts per million (ppm) relative to tetramethylsilane (TMS) as the internal standard. NMR data is presented as follows: Chemical shift, multiplicity (s = singlet, bs = broad singlet, d = doublet, t = triplet, dd = doublet of doublet, dt = doublet of triplet, m = multiplet and/or multiple resonances), coupling constant in Hertz (Hz), integration and assignment. All NMR signals were assigned on the basis of ^{1}H, ^{13}C, COSY, HSQC and HMBC experiments. Automatic column chromatography was performed on Biotage Isolera Spektra One, using SNAP cartridges 10-50g filled with normal silica (Biotage, 30-100 µm, 60 Å). TLC analysis was conducted on TLC Silicagel, 60, F254, Merck, with detection by UV absorption (254 nm) and staining.

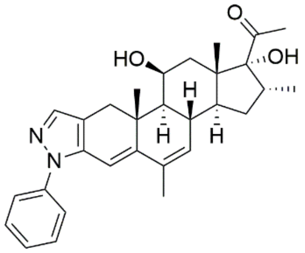

Structural analysis of hit molecule **NSC80997**. **TLC:** (50:50, EtOAc:Hept v/v) R_f = 0.35 **^{1}H NMR** (500 MHz, $CDCl_3$) δ 7.54 – 7.46 (m, 5H, H7&H24&H25&H27&H28), 7.37 – 7.33 (m, 1H, H26), 6.30 (d, J = 1.0 Hz, 1H, H11), 5.69 (s, 1H, H14), 4.46 (t, J = 3.1 Hz, 1H, H22), 3.12 (ddp, J = 11.0, 7.3, 4.0 Hz, 1H, H18), 2.99 – 2.95 (m, 1H, H5a), 2.92 (s, 1H, H34), 2.69 – 2.62 (m, 2H, H5b&H15), 2.27 (s, 3H, 3xH2), 2.05 (dd, J = 14.1, 3.7 Hz, 1H, H21a), 2.01 – 1.87 (m, 2H, H3&H17a), 1.85 (dd, J = 2.0, 1.3 Hz, 3H, 3xH31), 1.78 (s, 1H, H33), 1.52 – 1.41 (m, 3H, H21b&H17b&H16), 1.20 (s, 3Hx 3xH32), 1.15 (s, 3H, 3xH29), 0.92 (d, J = 7.2 Hz, 3H, 3x H30). **^{13}C NMR** (126 MHz, $CDCl_3$) δ 211.90 (C1), 147.78 (C12), 139.90 (C23), 138.28 (C7), 137.55 (C10), 132.47 (C14), 130.98 (C13), 129.36 (C24&C28), 127.12 (C26), 123.63 (C25&C27), 115.39 (C6), 106.52 (C11), 90.76 (C19), 68.27 (C22), 53.37 (C3), 49.78 (C20), 49.55 (C16), 40.16 (C21), 39.37 (C4), 35.67 (C18), 32.90 (C15), 32.54 (C17), 31.84 (C5), 27.98 (C2), 20.11 (C31), 20.02 (C32), 18.20 (C29), 15.09 (C30).

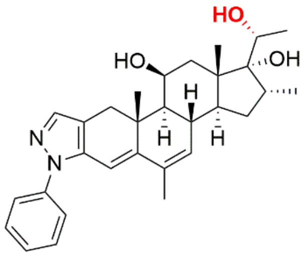

Synthesis of **NSC80997-KR.** The chemical procedure was adapted from [1]. Compound **NSC80997** (12.8 mg; 27.1 µmol) was fully dissolved in methanol (542 µL; 50 mM) by heating. The solution was then cooled to 0°C and then treated with sodium borohydride (1.5 mg; 40.6 µmol; 1.5 eq). After 3 hours additional sodium borohydride (3.1 mg; 81.2 µmol; 3 eq) was added and the mixture was stirred for an additional 1.5 hours. The solvent was removed by rotatory evaporation. Water (10 mL) was added and the product was extracted into ethyl acetate (5x 20 mL). The combined organic layers were dried over anhydrous sodium sulfate, filtered and concentrated *in vacuo*. The residue was purified by silicagel flash column chromatography (10% → 50% ethyl acetate in heptane) and lyophilized to afford **NSC80997-KR** (2.48 mg; 5.22 µmol; 19%). **TLC:** (60:40, EtOAc:Hept v/v) R_f = 0.25. **^{1}H-NMR** (500 MHz, $CDCl_3$) δ 7.55 – 7.46 (m, 5H, H24&H25&H27&H28&H7), 7.37 – 7.32 (m, 1H, H26), 6.30 (s, 1H, H11), 5.72 (s, 1H, H14), 4.44 (d, *J* = 3.1 Hz, 1H, H22), 4.03 (q, *J* = 6.4 Hz, 1H, H1), 2.99 (d, *J* = 15.2 Hz, 1H, H5a), 2.68 – 2.59 (m, 2H, H5b&H15), 2.03 (dq, *J* = 12.2, 5.2, 3.6 Hz, 1H, H18), 1.92 (t, *J* = 3.0 Hz, 2H, 2xH21), 1.85 (t, *J* = 1.7 Hz, 3H, 3xH31), 1.84 – 1.73 (m, 2H, H16&H17a), 1.48 – 1.40 (m, 2H, H3&H17b), 1.20 (s, 2H, 3xH32), 1.19 – 1.17 (m, 6H, 3xH2, 3xH29), 1.01 (d, *J* = 7.1 Hz, 3H, 3xH30). **^{13}C-NMR** (126 MHz, $CDCl_3$) δ 147.95 (C12), 139.99 (C23), 138.34 (C7), 137.61 (C10), 132.88 (C14), 130.80 (C14), 129.37 (C24&C28), 127.08 (C26), 123.65 (C25&C27), 115.51 (C6), 106.43 (C11), 84.51 (C19), 71.10 (C1), 68.75 (C22), 53.18 (C3), 49.00 (C20), 47.99 (C16), 42.54 (C21), 39.38 (C4), 35.79 (C18), 34.79 (C17), 32.91 (C15), 31.97 (C5), 20.18 (C31), 20.01 (C32), 19.56 (C2), 18.28 (C29), 16.65 (C30).

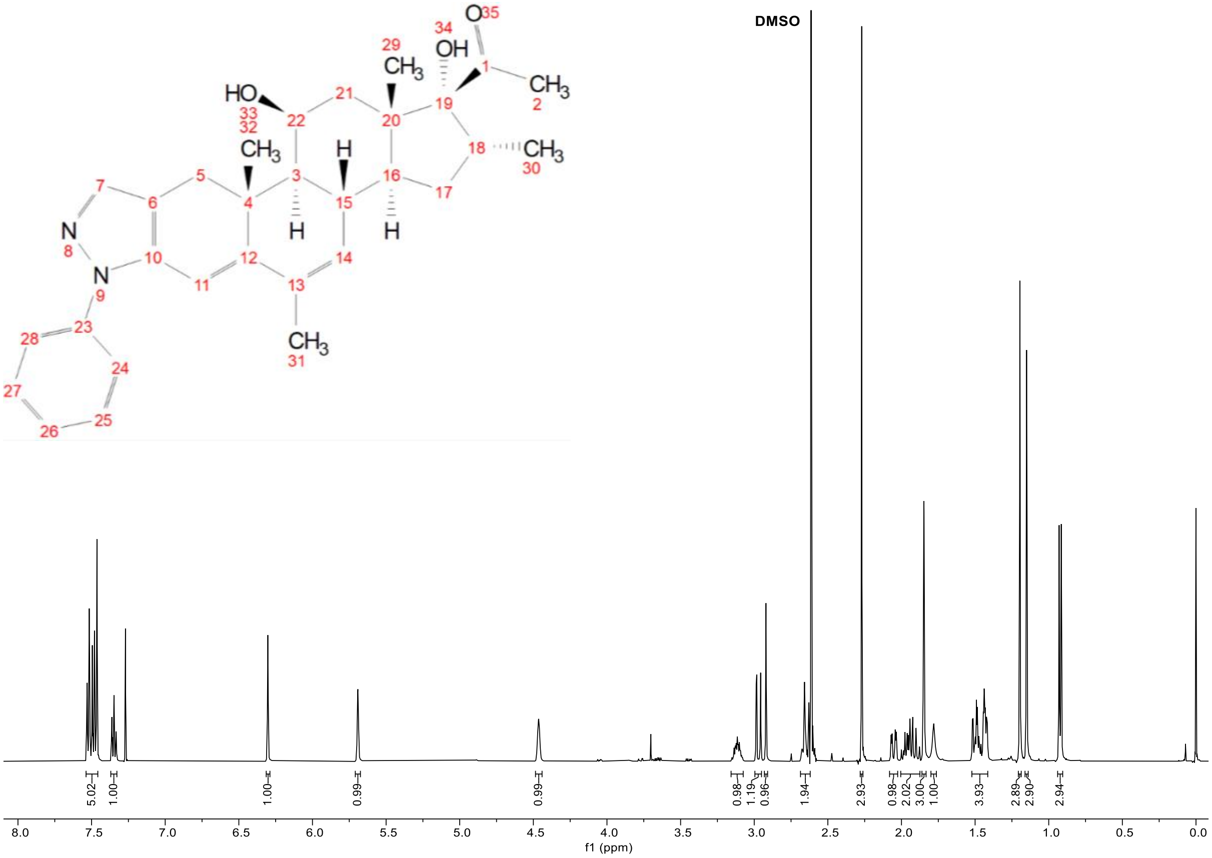
DMSO
f1 (ppm)

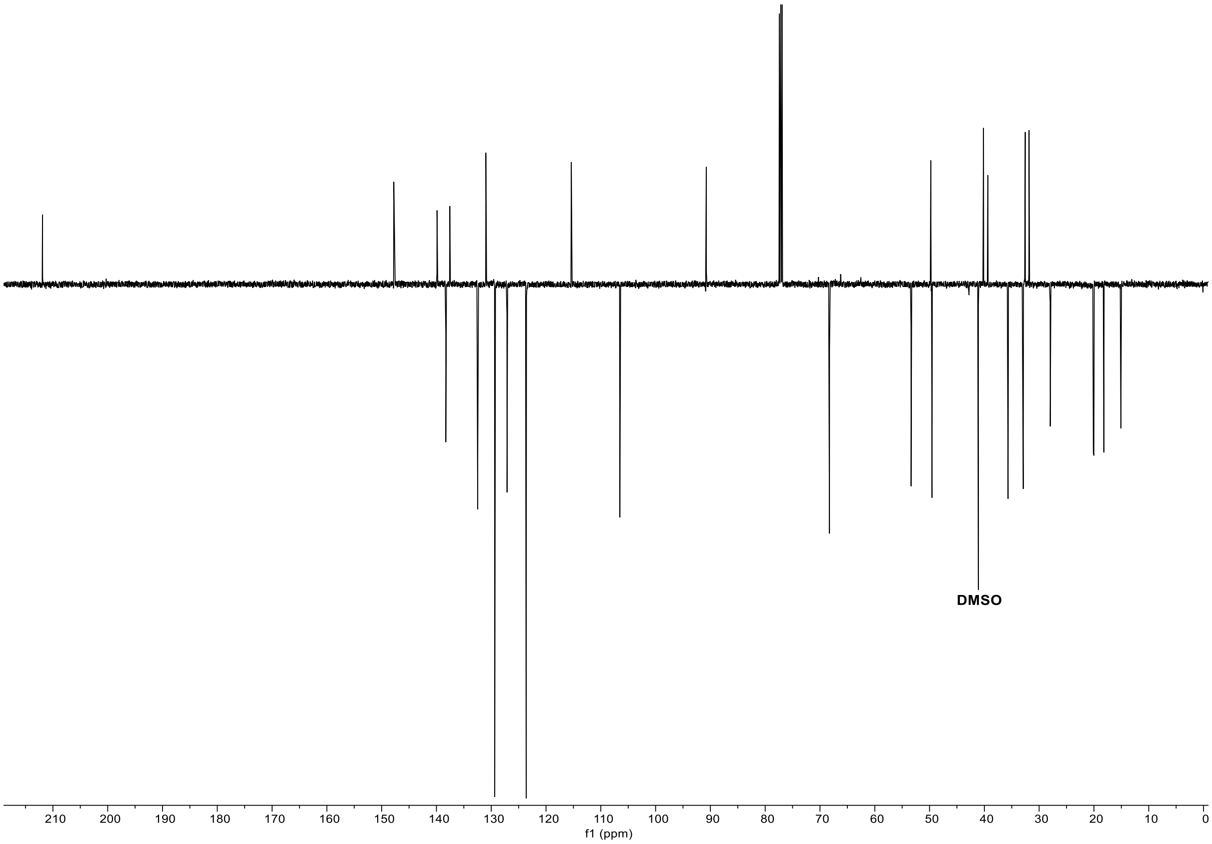
DMSO
f1 (ppm)

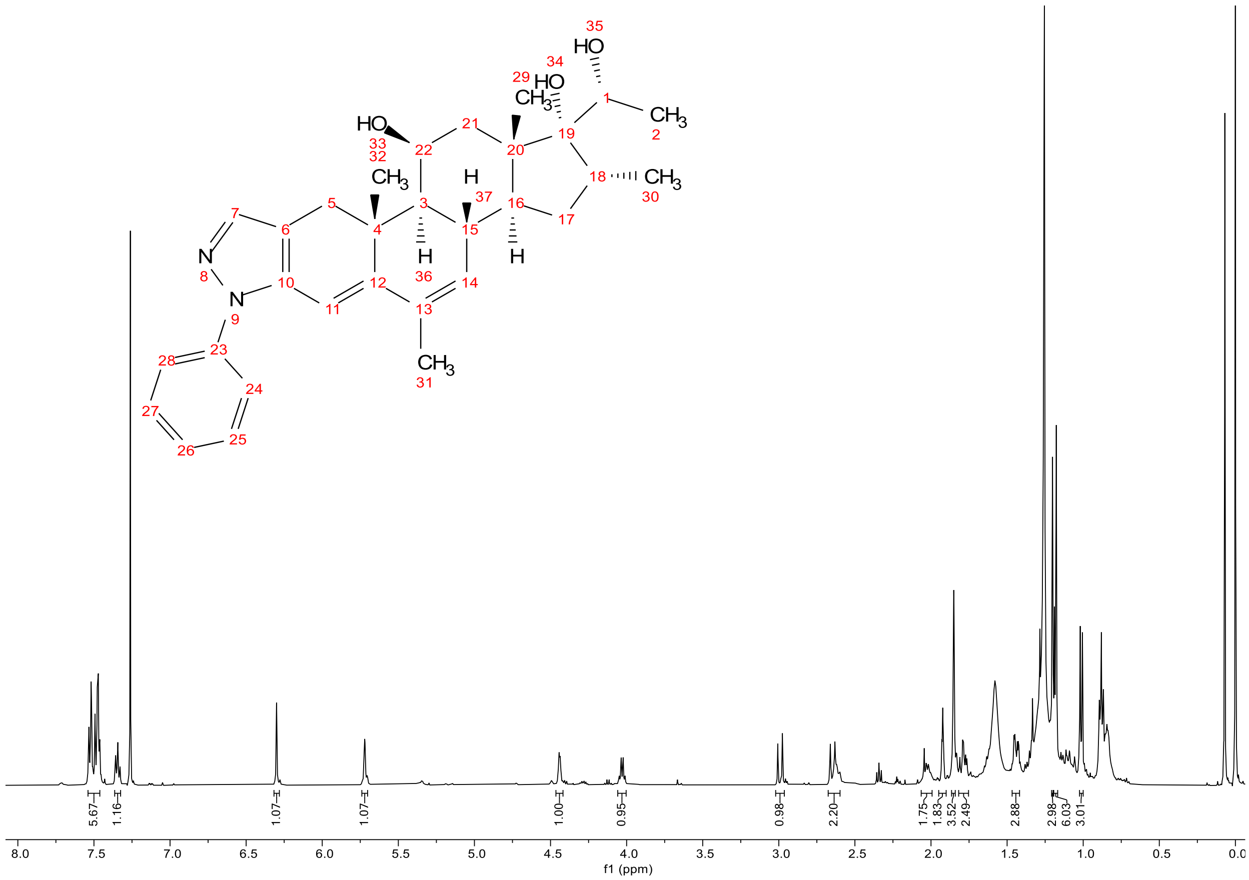

HO
HO
HO
CH3
CH3
CH3
CH3
CH3
H
H
H
N
N
5.67
1.16
1.07
1.07
1.00
0.95
0.98
2.20
1.75
1.83
3.52
2.49
2.88
2.98
6.03
3.01
8.0 7.5 7.0 6.5 6.0 5.5 5.0 4.5 4.0 3.5 3.0 2.5 2.0 1.5 1.0 0.5 0.0
f1 (ppm)

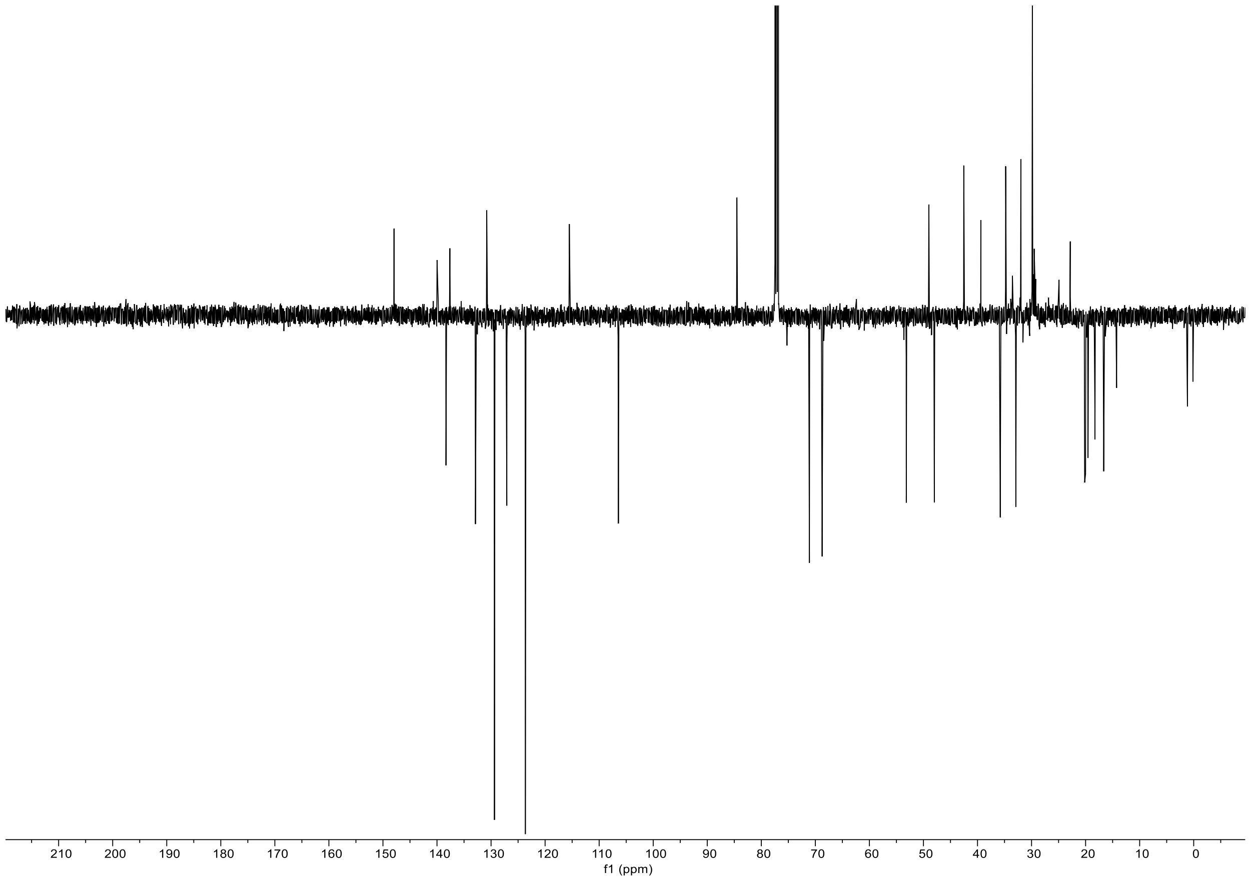

Supplementary Reference:

1. Ngo, A.H., Adams, M.J., and Do, L.H. (2014). Selective Acceptorless Dehydrogenation and Hydrogenation by Iridium Catalysts Enabling Facile Interconversion of Glucocorticoids. Organometallics *33*, 6742-6745. 10.1021/om5010258.
