# Supplementary material for: Identification of global inhibitors of cellular glycosylation
Source: Nat Commun. 2023 Feb 20;14:948. doi: 10.1038/s41467-023-36598-7 (PMC9941569; doi:10.1038/s41467-023-36598-7)
Supplement: Supplementary file 3 — Description to Additional Supplementary Files [file 41467_2023_36598_MOESM3_ESM.docx]

Description of Additional Supplementary Files

**Supplementary Movie 1-3.** Spinning-disk confocal microscopy of mScarlettGiantin-transfected HeLa cells treated with either Sup video 1) Vehicle (DMSO), Sup video 2) 10μM NSC80997 or Sup video 3) 10μM Brefeldin A. Videos are maximumintensity projections of image stacks acquired for a period of 1h (h:min:s:ms) from addition of drug or vehicle with a frame rate of 1 frames/min. Scale bars = 10 μm.
