# Supplementary material for: Identification of global inhibitors of cellular glycosylation
Source: Nat Commun. 2023 Feb 20;14:948. doi: 10.1038/s41467-023-36598-7 (PMC9941569; doi:10.1038/s41467-023-36598-7)
Supplement: Supplementary file 7 — Reporting Summary [file 41467_2023_36598_MOESM7_ESM.pdf]

nature portfolio

Corresponding author(s): Prof. Henrik Clausen
Associate prof. Yoshiki Narimatsu

Last updated by author(s): Dec 4, 2022

Reporting Summary

Nature Portfolio wishes to improve the reproducibility of the work that we publish. This form provides structure for consistency and transparency in reporting. For further information on Nature Portfolio policies, see our Editorial Policies and the Editorial Policy Checklist.

Statistics

For all statistical analyses, confirm that the following items are present in the figure legend, table legend, main text, or Methods section.

n/a	Confirmed	
☐	☒	The exact sample size (n) for each experimental group/condition, given as a discrete number and unit of measurement
☐	☒	A statement on whether measurements were taken from distinct samples or whether the same sample was measured repeatedly
☐	☒	The statistical test(s) used AND whether they are one- or two-sided<br>*Only common tests should be described solely by name; describe more complex techniques in the Methods section.*
☐	☒	A description of all covariates tested
☐	☒	A description of any assumptions or corrections, such as tests of normality and adjustment for multiple comparisons
☐	☒	A full description of the statistical parameters including central tendency (e.g. means) or other basic estimates (e.g. regression coefficient) AND variation (e.g. standard deviation) or associated estimates of uncertainty (e.g. confidence intervals)
☐	☒	For null hypothesis testing, the test statistic (e.g. F, t, r) with confidence intervals, effect sizes, degrees of freedom and P value noted<br>*Give P values as exact values whenever suitable.*
☒	☐	For Bayesian analysis, information on the choice of priors and Markov chain Monte Carlo settings
☒	☐	For hierarchical and complex designs, identification of the appropriate level for tests and full reporting of outcomes
☒	☐	Estimates of effect sizes (e.g. Cohen's d, Pearson's r), indicating how they were calculated

Our web collection on statistics for biologists contains articles on many of the points above.

Software and code

Policy information about availability of computer code

Data collection	Flow cytometry data was collected using the Sony SONY SA3800 software or BD Bioscience BDFACStation software (v8.0.1). For immunocytochemistry a Zeiss microscopy system and associated software was used. Spinning disk images were acquired on a Nikon Ti2 inverted fluorescence microscope using the NIS Elements AR 5.21.03. For protein gels and western blots an ImageQuant LAS 4000 system and associated software (GE Healthcare) were used. Mass spectrometry experiments were collected with an Orbitrap Fusion/Lumoms (Thermo) and Autoflex (Bruker). HPLC data was collected with Empower 3 Chromatography Data Software (Waters). Infection data was collected on an Incucyte S3 (Sartorius) using the onboard software.
Data analysis	FlowJo X 10.0.7r2 software for flow cytometry, ImageJ (NIH) Version 2.1.0/1.53c for immunohistochemistry, ImageQuant software (GE Healthcare) for protein gels and western blot analysis, FlexAnalysis (Bruker) and Proteome Dicoverer version 2.2 (Thermo Fisher) for mass spectrometry data analysis, Chromeleon 6.8 CHromatography Data system software (Thermo) for HPLC data analysis. Imaris 9.2.1 (Bitplane AG) software for colocalization analysis and timelapse imaging. Statistical analysis and graph preparation was performed using GraphPad Prism version 8.4.2 (GraphPad Software).

For manuscripts utilizing custom algorithms or software that are central to the research but not yet described in published literature, software must be made available to editors and reviewers. We strongly encourage code deposition in a community repository (e.g. GitHub). See the Nature Portfolio guidelines for submitting code & software for further information.

Data

Policy information about availability of data

All manuscripts must include a data availability statement. This statement should provide the following information, where applicable:

- Accession codes, unique identifiers, or web links for publicly available datasets
- A description of any restrictions on data availability
- For clinical datasets or third party data, please ensure that the statement adheres to our policy

All data generated or analyzed during this study is included in this article and supplementary information files. The mass spectrometry proteomics data has been deposited to the ProteomeXchange Consortium via the PRIDE repository with identifier PXD029623 [http://proteomecentral.proteomexchange.org/cgi/GetDataset?ID=PXD029623]. Source data are provided with this paper.

Human research participants

Policy information about studies involving human research participants and Sex and Gender in Research.

Reporting on sex and gender	Not applicable
Population characteristics	Not applicable
Recruitment	Not applicable
Ethics oversight	Not applicable

Note that full information on the approval of the study protocol must also be provided in the manuscript.

Field-specific reporting

Please select the one below that is the best fit for your research. If you are not sure, read the appropriate sections before making your selection.

☒ Life sciences ☐ Behavioural & social sciences ☐ Ecological, evolutionary & environmental sciences

For a reference copy of the document with all sections, see nature.com/documents/nr-reporting-summary-flat.pdf

Life sciences study design

All studies must disclose on these points even when the disclosure is negative.

Sample size	Sample size was chosen based on experience of the investigators with similar experiments conducted multiple times and previously published effect size (Narimatsu et al., Mol Cell 2019; Bull etl, PNAS. 2021.; Nason et al., Nat Commun 2021; Clausen et al. Cell (2020))
Data exclusions	No data was excluded from the manuscript
Replication	Flow cytometry analysis was performed multiple times (2-3 times) and all attempts at replication were successful. All SDS-PAGE and western blot experiments were carried out 2-3 times with similar results. Immunocytochemistry and live cell time lapse acquisition was performed independently 2-3 times for each experiment with similar results. Differential proteomic analysis was performed once, with biological triplicates in control and treatment group, with all attempts are replication being successful. Other mass spectrometry experiments were carried out twice with similar results. Viral infection assays were carried out twice in duplicates with similar results) and reported together.
Randomization	No group allocation was performed, randomization is irrelevant to the study.
Blinding	No group allocation was performed, blinding is irrelevant to the study.

Reporting for specific materials, systems and methods

We require information from authors about some types of materials, experimental systems and methods used in many studies. Here, indicate whether each material, system or method listed is relevant to your study. If you are not sure if a list item applies to your research, read the appropriate section before selecting a response.

Materials & experimental systems

n/a	Involved in the study
☐	☒ Antibodies
☐	☒ Eukaryotic cell lines
☒	☐ Palaeontology and archaeology
☒	☐ Animals and other organisms
☒	☐ Clinical data
☒	☐ Dual use research of concern

Methods

n/a	Involved in the study
☒	☐ ChIP-seq
☐	☒ Flow cytometry
☒	☐ MRI-based neuroimaging

Antibodies

Antibodies used	Commercial Antibodies:<br>Mouse anti-6xHis (R&D Systems) Cat: IC0501R; Biotinylated Lectins (Vector Labs); SiaFind Pan-Specific Lectenz and 2-3-specific Lectenz (Lectenz Bio) Cat: SK0501B and SK2301, respectively; Goat anti-mouse IgG AlexaFlour647 (Invitrogen) Cat: A21235; Rabbit Anti-Mouse Immunoglobulins Polyclonal Antibody FITC Conjugated (Dako) Cat: F031302; Anti-Glucocorticoid Receptor Antibody (Thermo Fisher) Cat: PA1-510A; Anti-Giantin antibody (Abcam) Cat: ab24586 or ab80864; Anti-TGN46 antibody (Abcam) Cat: ab50595; Mouse IgG Isotype Control (Invitrogen) Cat: 10400C; Anti-VSV-G antibody I1 (ATCC) Cat: CRL-2700; anti-Nucleocapsid antibody (GeneTex) Cat: GTX135357; Anti-Calnexin antibody (Abcam) Cat: ab92573; Anti- alpha Tubulin Antibody (Invitrogen) Cat: A11126; Anti-Bcop (Abcam) Cat: 2899; Anti-GM130 (BD-Biosciences) Cat: 610822; Anti-Tubulin (Sigma) Cat: T6199, Anti-AcTub (Sigma) Cat: T7451; CyTM3 AffiniPure Donkey Anti-Mouse IgG (ImmunoResearch) Cat: AB_2340813; Goat anit- mouse IgG, Alexa Flour 647 (Invitrogen by Thermo Fisher) Cat: A21235; Goat anti-mouse IgG, Alexa Flour 488 (Invitrogen by Thermo Fisher) Cat: A10680<br><br>In-house monoclonal antibodies:<br>Anti-GalNAc-T2 (UH2 6B7); Anti-GalNAc-T1 (UH3 4D8); anti-MUC1 (5E10); anti-Tn-MUC1 (5E5); anti-FXYD5 (NCC-M53); anti-Tn-FXYD5 (6C3); anti-Tn (1E3)
Validation	Commercially purchased antibodies against standard protein tags were validated on cell lines transfected with proteins with or without the relevant tag.<br>Anti-Glucocorticoid Receptor Antibody (Thermo Fisher) Cat: PA1-510A was validated in human samples in Melhelm et al. 2009 (DOI: 10.1158/1078-0432.CCR-08-2131)<br>Anti-Giantin antibody (Abcam) Cat: ab24586 was validated in human samples in Schumann B et al. Mol Cell (2020) (DOI: 10.1016/j.molcel.2020.03.030)<br>Anti-Giantin antibody (Abcam) Cat: ab80864 was validated in human samples in Wang et al. Nucleic Acids Res (2019) (DOI: 10.1093/nar/gkz659).<br>Anti-TGN46 antibody (Abcam) Cat: ab50595 was validated in human samples in Liu Y et al. Nat Commun (2020) (DOI:10.1038/s41467-020-14470-2).<br>Anti-VSV-G antibody I1 (ATCC) Cat: CRL-2700 and anti-Nucleocapsid antibody (GeneTex) Cat: GTX135357 are validated in Clausen et al. Cell (2020) (DOI: 10.1016/j.cell.2020.09.033).<br>Anti-Calnexin antibody (Abcam) Cat: ab92573 is validated in Horner SM et al. Proc Natl Acad Sci U S A (2011) (DOI: 10.1073/pnas.1110133108)<br>Anti- alpha Tubulin Antibody (Invitrogen) Cat: A11126 was validated in Roeles J, Tsiavaliaris G. Nat Commun. (2019) doi: 10.1038/s41467-019-12674-9.<br>Anti-AcTub (Sigma) Cat: T7451was validated in Piperno G, et al. J Cell Biol. (1987) doi: 10.1083/jcb.104.2.289.<br>Anti-Bcop (Abcam) Cat: 2899 was validated in Sasako et al. Nat Commun. (2019) doi: 10.1038/s41467-019-08591-6.<br>Anti-GM130 (BD-Biosciences) Cat: 610822 was validated in Marra P, et al. Nat Cell Biol. (2001) doi: 10.1038/ncb1201-1101. PMID: 11781572.<br>Anti-Tubulin (Sigma) Cat: T6199 was validated in Breitling, F., and Little, M., J. Molec. Biol., 189, 367-370 (1986).Wolff, A., et al., Biol. Cell, 63, 319-326 (1988).Serrano, L., et al., Anal. Biochem., 159, 253-259 (1986)<br>Mouse anti-6xHis antibody (R&D Systems) is validated in Narimatsu et al., Mol Cell 2019. Biotinylated lectins (Vector Labs) and SiaFind Pan-specific and 2-3-specific Lenctenz (Lectenz Bio) are validated in Bull etl, PNAS. 2021.

Eukaryotic cell lines

Policy information about cell lines and Sex and Gender in Research

Cell line source(s)	HEK293 (ECACC 85120602); HEK293 6E (National Research Council, Canada); CHOZN GS-/- (Sigma); MCF7 (ATCC: HTB-22); HeLa (ATCC: CCL-2); SH-SY5Y (ATCC: CRL-2266); AGS (ATCC: CRL-1739); Vero-E6 (ATCC: CRL-1586); A549 (ATCC: CRM-CCL-185); Vero-TMPRSS2 (SEKISUI, XenoTech); Caco2 (ATCC: HTB-37); Huh 7.5 (Apath, LLC)
Authentication	No specific authentication of cell lines was used apart from separate handling of original obtained vials throughout the entire project. Each individual engineered HEK293 cell clone were confirmed multiple times by HEK293 gene specific IDAA and Sanger sequencing in the target gene area.
Mycoplasma contamination	A representative set of growing cells in the lab is selected randomly and subjected to mycoplasma screening bi-monthly, and no infections has been found in the last 10 years.
Commonly misidentified lines<br>(See ICLAC register)	None of the cell lines used are were found in the ICLAC database

Flow Cytometry

Plots

Confirm that:

☒ The axis labels state the marker and fluorochrome used (e.g. CD4-FITC).

☒ The axis scales are clearly visible. Include numbers along axes only for bottom left plot of group (a 'group' is an analysis of identical markers).

☒ All plots are contour plots with outliers or pseudocolor plots.

☒ A numerical value for number of cells or percentage (with statistics) is provided.

Methodology

Sample preparation	Cells treated with compounds or DMSO control were stained with biotinylated lectins (Vector Laboratories) or mouse monoclonal antibodies against Tn (1E3)41,92, Tn-MUC1 (5E5)58, FXYD5 (6C5 and NCC-MC53)57 diluted in PBA (PBS with 1% (w/v) BSA) for 1h at 4°C. For SARS-CoV-2 spike protein binding, NSC80997, DMSO control or Heparinase mix (2.5 mU/mL HSase II, and 5 mU/mL HSase III; IBEX) treated cells were incubated with recombinant SARS-CoV-2 biotinylated spike protein S1/S2 (20 µg/mL) for 30 min at 4°C. Spike protein was produced and biotinylated as previously described 64. Cells were washed with PBA and incubated with streptavidin conjugated to Alexa Fluor 488 or 647 (Invitrogen), FITC-conjugated rabbit anti-mouse immunoglobulins (Dako), or Alexa Fluor 647-conjugated goat-anti mouse IgG (Thermo Fisher), respectively. Cells were washed twice and resuspended in PBA for analysis
Instrument	A SA3800 spectral analyzer (SONY) or a FACSCalibur instrument (BD Bioscience).
Software	Data was analyzed using FlowJo software (FlowJo, LCC).
Cell population abundance	Not applicable. Gating was performed only to exclude dead cells and doublets
Gating strategy	Dead cells were excluded based on forward and side scatter area (FSC-A and SSC-A) parameters. Doublets were excluded based on FSC-H (height) and FSC-W (width) parameters

☒ Tick this box to confirm that a figure exemplifying the gating strategy is provided in the Supplementary Information.
